# Benefits of a *Bacillus* probiotic to larval fish survival and transport stress resistance

**DOI:** 10.1038/s41598-019-39316-w

**Published:** 2019-03-20

**Authors:** Andrea M. Tarnecki, Marzie Wafapoor, Remy N. Phillips, Nicole R. Rhody

**Affiliations:** 1 Mote Marine Laboratory, Marine Immunology Program, 1600 Ken Thompson Parkway, Sarasota, FL 34236 USA; 2 Marine Science Center, Northeastern University, 430 Nahant Road, Nahant, Massachusetts 01908 USA; 3 Mote Marine Laboratory, Marine and Freshwater Aquaculture Program, 874 WR Mote Way, Sarasota, FL 34240 USA

## Abstract

The need for sustainable bacterial management approaches in aquaculture is crucial for advancement of the industry. Probiotics are a promising strategy as evidenced by benefits demonstrated in intensive larviculture of various marine fish species. In this study we investigate the effects of a mixed *Bacillus* species (*B. licheniformis* and *B. amyloliquefaciens*) probiotic on rearing of larval common snook (*Centropomus undecimalis*). Experimental treatments included (1) probiotics supplemented to the water and live feed, (2) probiotics supplemented to the water only, and (3) no probiotic controls. Data from two separate trials indicated up to 2.5 times higher survival with probiotic addition, as well as 20% higher survival 7 days following a transport event. These benefits were not explained by faster growth, measured water quality parameters, or innate immune enzyme activities. Microbiota analysis indicated the importance of system stabilization prior to larval stocking to improve rearing success and probiotic performance. ied Potential probiotic benefits include accelerated gastrointestinal tract development, enhanced immunity, inhibition of opportunistic bacteria, and improvements to water quality parameters. Results suggest this probiotic should be tested in other marine fish species in order to reduce larval rearing bottlenecks.

## Introduction

As the human population grows and demand for seafood rises, total fishery production worldwide is expected to increase 17% by 2025^1^. A majority of wild fish stocks are currently fished at biologically unsustainable levels or overfished without expansion potential, and aquaculture already provides over half of all seafood consumed; thus, aquaculture will account for a majority of increased seafood production^1^. Despite its potential, high mortality of fishes in larval stages remains a bottleneck for this industry. Varying survival and reproducibility between tanks originating from the same spawning event suggest that detrimental host-microbe interactions are responsible for these inconsistencies^2^. Intensive production systems including recirculating aquaculture systems (RAS) support the proliferation of bacterial pathogens and opportunists due to the excess input of organic matter via feed, feces, and dead larvae^2^, leaving fish larvae without fully developed immune systems vulnerable to these bacteria. Furthermore, water in RAS is often treated via ozonation and UV sterilization prior to the addition of larvae, which may further disrupt complex microbial interactions and benefit opportunistic pathogen proliferation^3^. Larval fish ingest microbes by drinking before exogenous feeding begins; therefore, the microbiota and water quality of the rearing environment impacts health and survival during these stages, and a lack of a stable, healthy microbiota prior to addition of larvae likely plays a large role in the survival variability seen in rearing systems.

Traditional bacterial management strategies in animal production systems have employed the use of chemicals, including antibiotics, to treat and prevent diseases. However, emergence of antibiotic-resistant pathogens and knowledge of the effect of these chemicals on structure of the commensal microbiota in the water column and gastrointestinal tract of fishes prompted alternative, sustainable strategies aimed at bacterial management in aquaculture^4^. One such strategy is the use of probiotics which are live, beneficial bacteria supplemented to the fish or water to improve water quality, digestion, and immune function. Probiotic bacteria colonize the larval fish and improve health via competitive exclusion and production of antimicrobial compounds against pathogens and opportunists^5^. Stimulation of the innate immune system of larval fish by probiotics provides rapid activation of antigens that improves survival and resistance to pathogens^6^. Important in enhancement of innate immunity is the up-regulation of certain enzymes that allow the organism to cope with cellular stress. Some probiotic bacteria, such as *Bacillus*, boost production of these vital enzymes.

*Bacillus* probiotics are promising for commercial production as they are spore-forming bacteria, allowing for a greater shelf life^7^. Some species of *Bacillus* control the growth of opportunistic pathogens^8^ and produce anti-viral compounds^9^. Furthermore, probiotic addition through live feeds, such as rotifers, *Artemia*, and copepods have resulted in improved fish health by increasing survival, growth rates, and enzymatic immune responses^10–12^. However, the mechanisms behind these benefits remain poorly understood. Therefore, studies investigating and characterizing the effect of probiotic addition on the microbiota, innate immunity, and development of fishes during larviculture are necessary for optimization of these alternative bacterial management strategies.

Common snook *Centropomus undecimalis* are a model organism for intensive fish production as they represent a popular food fish in Central and South America and a high value commercial sport fishery in the US. Currently, snook production in the US is focused on fisheries restoration efforts to restore stock losses due to overfishing, weather phenomenon (i.e., cold temperatures), and habitat loss^13^. However, as with other marine species, high mortality in the first 30 days post hatch continues to be a major bottleneck in snook aquaculture production^14^.

To that end, the objective of this study was to test the efficacy of two probiotic *Bacillus* strains during the common snook larval rearing phase of production.

## Methods

All procedures performed were in accordance with the ethical standards of the institution outlined in Mote Marine Laboratory’s Animal Welfare Assurance (A4219-01). All experimental protocols were approved by Mote Marine Laboratory’s Animal Care and Use Committee (IACUC Approval No. 17-10-KM1).

Two separate trials were conducted, with Trial 1 occurring in 2015 and Trial 2 in 2017.

### Egg and larvae collection

Snook broodstock were held in environmentally controlled indoor recirculating tank systems and spawned using routine methods^15^. Collected eggs were distributed into black, conical, 100 L hatching tanks (28 °C; one hatcher in Trial 1, two hatchers in Trial 2) with a flow rate of 3 L min^−1^. Eggs from Trial 1 were stocked at approximately 3,000 eggs L^−1^, for a total egg count of 300,000. Eggs hatched 16 h post-fertilization with a hatch rate of 80.4%. A total of 682,000 eggs were collected for Trial 2 and stocked at a stocking density of 3,410 eggs L^−1^. Eggs hatched 15 h post-fertilization with hatch rates for the two hatchers of 57.6 and 62.5%. Hatched larvae were volumetrically stocked at 100 larvae L^−1^ into three separate recirculating systems.

### Experimental design

Each experimental system (n = 3) consisted of a sump and six 100 L replicate tanks for a total volume of 1,155 L recirculating seawater. All recirculating systems were equipped with a solids filter, bio-filtration and UV sterilization. LED lights were set at a photoperiod of 12 L:12 D. A multiparameter water quality meter was used daily to test and maintain the following: salinity (35 ppt), temperature (28 °C), pH (8.4–8.5), and dissolved oxygen (D.O. 4–7 mg mL^−1^). Ammonia, nitrite, and nitrate were monitored weekly using UV/Vis spectroscopy. The flow rate was initially set at 500 mL min^−1^ and increased to 1 L min^−1^, at Day 10 in Trial 1 and Day 4 in Trial 2, for the remainder of the study.

### Probiotic administration

The probiotic mix contained one proprietary strain each of *Bacillus licheniformis* and *Bacillus amyloliquefaciens* (1:1) at 1 × 10^10^ CFU g^−1^. Growth on 5% sheep’s blood nutrient agar at 37 °C for 24 h indicated strains were non-hemolytic. Experimental treatments included (1) PBWF = probiotic added to water and live feed (rotifer) cultures; (2) PBWO = probiotic added to water only; (3) CONT = control with no probiotic added. The targeted probiotic concentration was 1.1 × 10^5^ CFU mL^−1^. In Trial 1, probiotic was added to the systems immediately prior to larval stocking, whereas Trial 2 systems received probiotics three times per week for five weeks prior to larval stocking in order to meet the desired concentration, confirmed by plate counts on marine agar.

### Live feed preparation

Probiotic was added to the concentration listed above only to live rotifer cultures for the PBWF treatment. Rotifers for all treatments were enriched with Algamac 3050 twice a day corresponding with morning and afternoon feedings. During Trial 1, larvae were fed rotifers from 2–27 days post hatch (dph) and co-fed *Artemia* starting at 21 dph for the remainder of the trial, concluding at 28 dph. Improvements in culture technologies for common snook during the two years between trials altered the feeding strategy for Trial 2, during which larvae were fed rotifers from 2–19 dph and co-fed *Artemia* and micro diet starting at 11 dph for the remainder of the trial, concluding at 26 dph.

### Larval morphometrics and survival

For standard length measurements, 50 larvae were collected from hatchers and 5 larvae per experimental tank were sampled at each specified day. Sampling days during Trial 1 occurred on 0, 3, and 14 dph, whereas larvae were additionally sampled at 1, 2, and 7 dph during Trial 2. Yolk sac and oil globule measurements were taken from hatch (0 h) to 48 hours post hatch (hph) during Trial 2 only. Yolk sac and oil globule volumes (mm^3^) were calculated as previously described^16^. Larvae were photographed using an Olympus BX53 microscope fitted with a DP-72 digital camera. Standard length, oil globule diameter, and yolk sac lengths and height were measured using Olympus CellSens version 1.15 imaging software. On the final day of each trial, standard lengths of 10 larvae per tank were measured using a digital caliper. In Trial 2, an additional 30 larvae per tank were obtained for dry weight measurements.

At the conclusion of the trials, larvae from each tank were counted to determine total survival. Final mean percent larval survival was calculated assuming an initial stocking density of 10,000 larvae per tank.

### Larval transport study

Following survival counts, 500 larvae from each treatment (n = 3) were stocked into four separate oxygenated bags (125 larvae per bag, 10 L^−1^ water) without probiotic. Trial 1 larvae were transported to Riverview High School (approximately 20 miles), whereas Trial 2 larvae were driven around our facility in a mock transport event (30 minutes). Water samples were taken from each transport bag (n = 12) prior to stocking the larvae and again at the end of the transport. Water quality (D.O., salinity, temperature, pH) and water chemistry (NH_3_-N) parameters were measured in each bag and were not significantly different among treatments pre or post transport. Oxygen was maintained between 6 and 8 mg L^−1^ and pH ranged from 7.8–8.0. Ammonia (NH_3_-N) measured 0.01 mg L^−1^ in all transport bags at the start of each trial and between 0.05 and 0.08 mg L^−1^ at the end. Surviving larvae were counted and set up in new tank systems without probiotic. Mean percent larval survival was determined immediately post-transport and again 7 days post-transport.

### Innate immune activity

Initial and final larval samples were taken from the hatchers (4 replicates each) and experimental tanks (1 replicate per tank), respectively. Each replicate consisted of 25 larvae (with the exception of the final sampling in Trial 1, during which 10 larvae were sampled per treatment tank). Larvae were homogenized in 750 µL sterile phosphate buffered saline using molecular biology grade 1.5 mm high impact zirconium beads in a three-tube bead homogenizer at max speed for 3 mins. Debris was removed by centrifugation at 10,000 × *g* for 5 min. Supernatant was stored at −80 °C until further analysis. Total protein was determined using the method established by Bradford^17^. Superoxide dismutase (SOD) activity was determined using the Superoxide Dismutase Assay kit (Cayman Chemical, Ann Arbor, MI), and lysozyme (LYS) activity was determined using the EnzChek™ Lysozyme Assay Kit (Thermo Fisher Scientific, Waltham, MA) according to manufacturers’ instructions. Alkaline phosphatase (ALP) (Trial 2 only) was determined using the protocol described by Ross *et al*.^18^. Immune enzyme activities were standardized to the total amount of protein, yielding final activities in U mg^−1^ total protein.

### Microbiota

Replicates of 25 larvae each were removed from the hatchers (4 replicates from the hatcher in Trial 1, 2 replicates from each hatcher in Trial 2) and rinsed with sterile water to remove transient, water-associated bacteria. Additionally, replicates of water (250 mL in Trial 1, 100 mL in Trial 2) were filtered through a 0.2 micrometer polycarbonate membrane filter to collect microorganisms. Larvae (25 larvae) and water (250 mL or 100 mL) were subsequently sampled from each experimental tank at each of the following days: 7, 14, and 28 or 26 dph. An additional water sample was processed from each experimental tank immediately prior to stocking of larvae (Day 0). Rinsed larvae and water filters were stored at −80 °C prior to DNA extractions.

Sequencing protocols differed between trials due to evolving access to resources. In Trial 1, DNA extractions were performed using the QIAamp DNA Microbiome kit (QIAGEN, Germantown, MD), whereas Trial 2 used the PowerSoil® DNA Isolation Kit (Mo Bio Laboratories, Carlsbad, CA, USA). In Trial 1, 16S MetaVx™ Mammalian sequencing was performed across the V3 and V4 hypervariable regions using proprietary primers and PCR protocols at GENEWIZ, Inc. (South Plainfield, NJ). In Trial 2, the bacterial 16S rRNA genes (V4 variable region) were amplified as previously described^14^. Sequence data from both trials were processed according to the MiSeq Illumina SOP protocol using Mothur v.1.35.1^19^, removing sequences less than 150 base pairs. Operational taxonomic units (OTUs) were defined by clustering filtered sequences at 97% similarity. OTUs were taxonomically classified using the SILVA database^20^ and again using the Greengenes database^21^ to allow for predictive functional profiling. Permutational analysis of variance (PERMANOVA) was used to compare microbiota between sample types (water versus larvae) and treatments (PBWF, PBWO, CONT) in Primer v6^22^. Predicted functions of the microbiota were analyzed using phylogenetic investigation of communities by reconstruction of unobserved states (PICRUSt)^23^ in Galaxy (http://huttenhower.sph.harvard.edu), with accuracy of predictions assessed using the weighted nearest sequenced taxon index (NSTI). Mothur was used to calculate the linear discriminant analysis effect size (LEfSe)^24^ to identify OTUs responsible for community differences between treatments.

### Statistical analysis

All statistical analyses were performed using R studio version 0.99.903. One-way ANOVAs followed by Tukey HSD post-hoc tests were used to examine differences in overall morphometrics, survival, and immune parameter data between treatments. Data not transformable to meet assumptions were analyzed using Kruskal-Wallis tests followed by Wilcoxon rank sum post-hoc tests.

## Results

### Water quality

Mean water quality (± SD) parameters during both trials were maintained within acceptable limits (Table 1), and water quality during Trial 2 was similar to that seen in Trial 1.

**Table 1:** Final water quality (average ± SD) for each treatment in two probiotic trials. CONT, control; PBWO, probiotics in water only; PBWF, probiotics in water and live feed.

Trial	Treatment	Temperature (°C)	Salinity (ppt)	Dissolved oxygen (mg L^−1^)	pH
Trial 1	CONT	28.4 ± 0.34	35.3 ± 0.47	4.7 ± 0.62	8.4 ± 0.11
	PBWO	28.3 ± 0.36	35.1 ± 0.92	4.3 ± 0.59	8.4 ± 0.11
	PBWF	28.3 ± 0.33	35.1 ± 0.46	4.2 ± 0.39	8.4 ± 0.14
Trial 2	CONT	28.8 ± 0.02	35.6 ± 0.04	7.1 ± 0.61	8.5 ± 0.02
	PBWO	28.8 ± 0.01	34.5 ± 0.14	7.1 ± 0.40	8.6 ± 0.01
	PBWF	28.7 ± 0.01	35.7 ± 0.26	6.0 ± 0.09	8.6 ± 0.01

### Morphometrics and survival

Yolk sac and oil globule volume resorption as measured in Trial 2 occurred rapidly within the first 24 hph, with yolk sacs depleting more rapidly than oil globule volumes across all three treatments (Table 2). Yolk sac volume was significantly higher in initial (Hatcher) larvae than all three treatments at 24 hph (χ^2^ = 117.85, df = 3, p < 0.001). Oil globule volume differed significantly among treatments after 24 hph (χ^2^ = 48.86, df = 3, p < 0.001) when PBWF larvae had higher mean oil globule volumes than CONT larvae (Wilcoxon rank sum, p < 0.05).

**Table 2:** Oil globule and yolk sac volumes (average ± SD) for probiotics Trial 2. Letters denote significant difference (p < 0.05). Hatcher, initial larvae taken from hatcher tanks; CONT, control; PBWO, probiotics in water only; PBWF, probiotics in water and live feed.

Hours post hatch (hph)	Treatment	Oil globule volume (mm^3^)	Yolk sac volume (mm^3^)
0	Hatcher	0.0047 ± 0.0009	1.398 ± 0.533
24	CONT	0.0038 ± 0.0008 b	0.140 ± 0.035
	PBWO	0.0046 ± 0.0013 ab	0.153 ± 0.064
	PBWF	0.0052 ± 0.0014 a	0.145 ± 0.038
48	CONT	0.0007 ± 0.0002	0.022 ± 0.005
	PBWO	0.0008 ± 0.0004	0.022 ± 0.007
	PBWF	0.0008 ± 0.0004	0.026 ± 0.006

**Figure 1:**
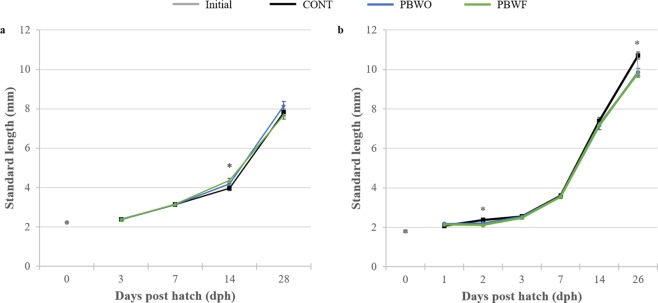
Standard length (mm, ±SE) of common snook larvae during two probiotic trials. Asterisks represent significant differences (p < 0.05). (**a**) Trial 1; (**b**) Trial 2.

In Trial 1, significant differences in larval standard lengths among treatments were detected only at 14 dph (χ^2^ = 17.398, df = 2, p < 0.001; Fig. 1a), at which time PBWF larvae were significantly longer than CONT larvae. In Trial 2, CONT larvae were significantly longer than probiotic-treated larvae at 2 dph (χ^2^ = 25.55, df = 2, p < 0.001, Fig. 1b) and 26 dph (ANOVA, F_2,157_ = 6.83, p < 0.05). CONT larvae also had significantly higher body mass than probiotic-treated larvae (ANOVA, F_2,157_ = 10.279, p < 0.001; TukeyHSD, p < 0.05; Table 3).

**Table 3:** Larval dry weights (average ± SD) for probiotics Trial 2. Dry weights were taken at 26 days post hatch. Letters denote significant differences (p < 0.05). CONT, control; PBWO, probiotics in water only; PBWF, probiotics in water and live feed.

Treatment	Dry Weight (µg)
CONT	0.0050 ± 0.0019 a
PBWO	0.0036 ± 0.0016 b
PBWF	0.0037 ± 0.0018 b

**Figure 2:**
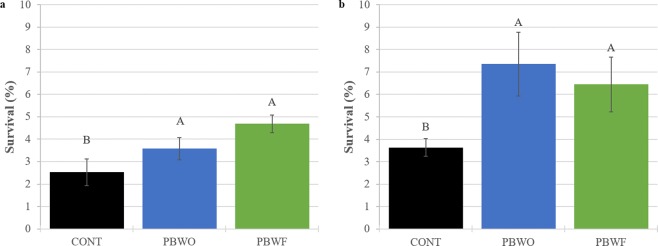
Percent survival (± SE) of common snook larvae treated with probiotics for 28 days (Trial 1) and 26 days (Trial 2). Letters denote significant differences (p < 0.05). (**a**) Trial 1; (**b**) Trial 2. CONT, control; PBWO, probiotics in water only; PBWF, probiotics in water and live feed.

Mean percent larval survival was significantly higher in PBWO and PBWF treatments than CONT at the conclusion of Trial 1 (28 dph) (ANOVA, F_2,15_ = 3.83, p < 0.05; Fig. 2a) and Trial 2 (26 dph) (ANOVA, F_3,12_ = 31.83, p < 0.05; Fig. 2b). In Trial 2, probiotic-treated larvae had 2.5 times higher average survival than CONT larvae.

### Transport study

**Figure 3:**
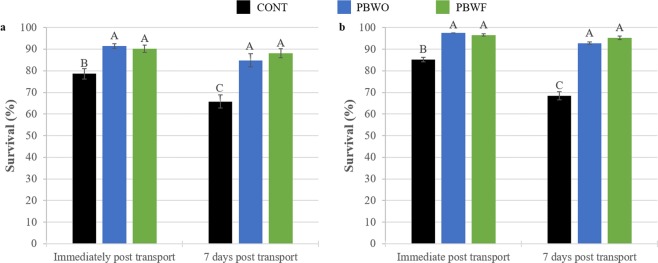
Percent survival (± SE) of common snook larvae after transport following experimental probiotics trials. Letters denote significant differences (p < 0.05). (**a**) Trial 1; (**b**) Trial 2. CONT, control; PBWO, probiotics in water only; PBWF, probiotics in water and live feed.

Transport results were nearly identical between trials (Fig. 3). Probiotic-treated larvae had approximately 10% higher survival immediately following transport and 20% higher survival one week after transport as compared to CONT larvae (Trial 1 ANOVA, F_2,18_ = 2.041, p < 0.05; Fig. 3a; Trial 2 ANOVA, F_2,18_ = 2.1.35, p < 0.001; Fig. 3b).

### Innate immunity

No significant differences were detected in measured innate immune enzyme activities (Table 4). However, SOD activity trended towards being higher in treated larvae than CONT in both trials, whereas LYS activity demonstrated the opposite trend. ALP activity was highest in CONT larvae in Trial 2.

**Table 4:** Innate immune enzyme activities (U mg^−1^) (average ± SD) for two probiotics trials. Activities were measured at 28 days post hatch in Trial 1 and 26 days post hatch in Trial 2. CONT, control; PBWO, probiotics in water only; PBWF, probiotics in water and live feed.

Trial	Treatment	Superoxide dismutase (SOD)	Lysosyme (LYS)	Alkaline phosphatase (ALP)
Trial 1	CONT	2.61 ± 0.25	2.38 ± 1.07	—
	PBWO	4.69 ± 0.68	1.87 ± 0.76	—
	PBWF	5.76 ± 0.52	1.75 ± 0.72	—
Trial 2	CONT	0.84 ± 0.43	9.80 ± 2.32	0.087 ± 0.07
	PBWO	2.52 ± 1.30	6.83 ± 2.97	0.042 ± 0.01
	PBWF	1.05 ± 0.28	8.39 ± 2.95	0.058 ± 0.07

### Microbiota

#### Microbiota structure

**Figure 4:**
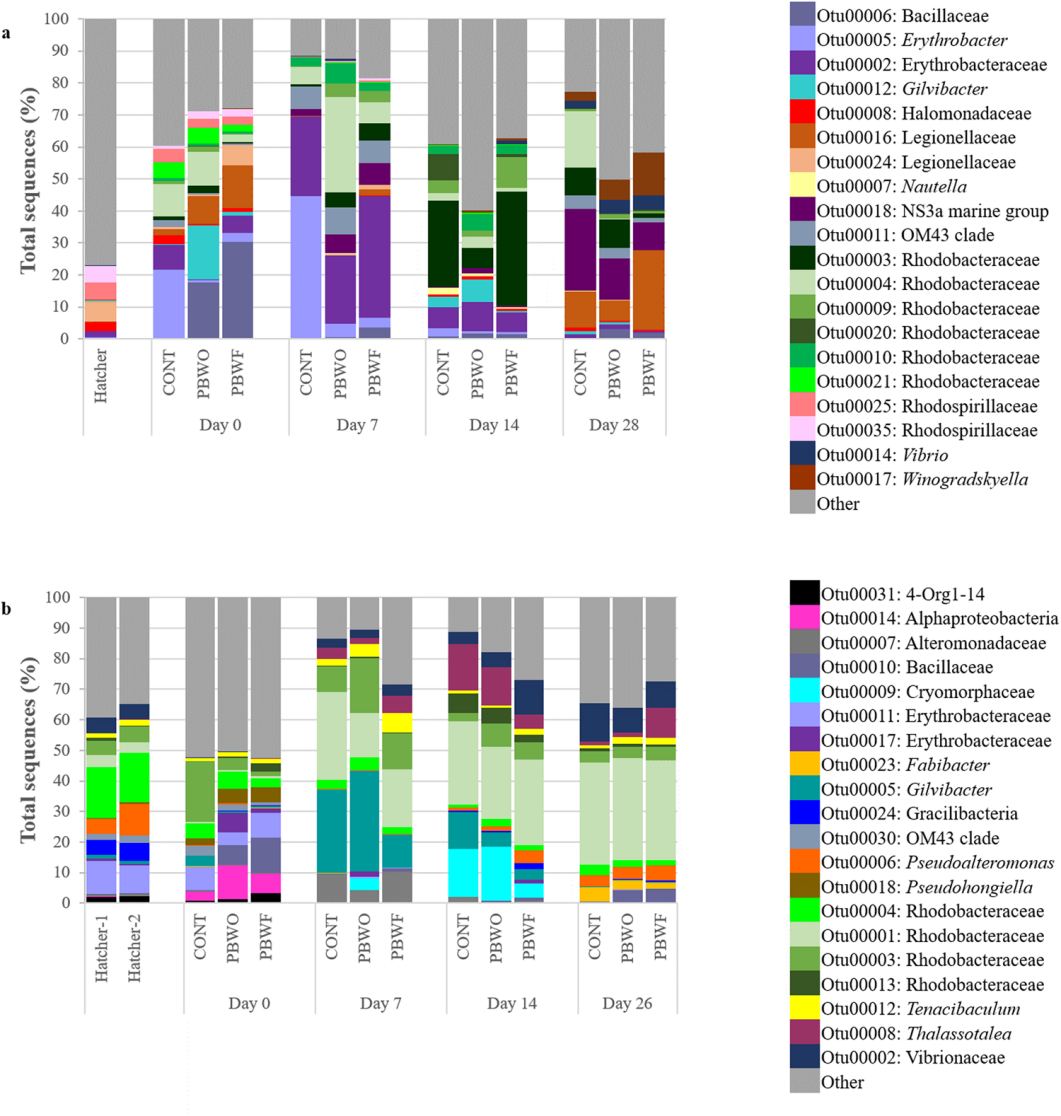
Relative abundances of the 20 most abundant OTUs identified in the water microbiota during probiotic trials with common snook larvae. (**a**) Trial 1; (**b**) Trial 2. Hatcher, water sampled from the egg hatcher; CONT, control; PBWO, probiotics in water only; PBWF, probiotics in water and live feed.

The 20 most dominant OTUs (shown at the taxonomic level) of the water microbiota for each trial are shown in Fig. 4. In both trials, Bacillaceae (likely representing the probiotic) had the highest relative abundance in treatment systems at Day 0, and these relative abundances decreased over time. OTUs identified as Erythrobacters were dominant at Day 0 in both trials and remained abundant until Day 14 in Trial 1, whereas they were largely absent by Day 7 in Trial 2. Rhodobacters were abundant in both trials throughout the sampling period. Other taxa abundant in both trials included *Gilvibacter*, the OM43 clade, and Vibrios.

**Figure 5:**
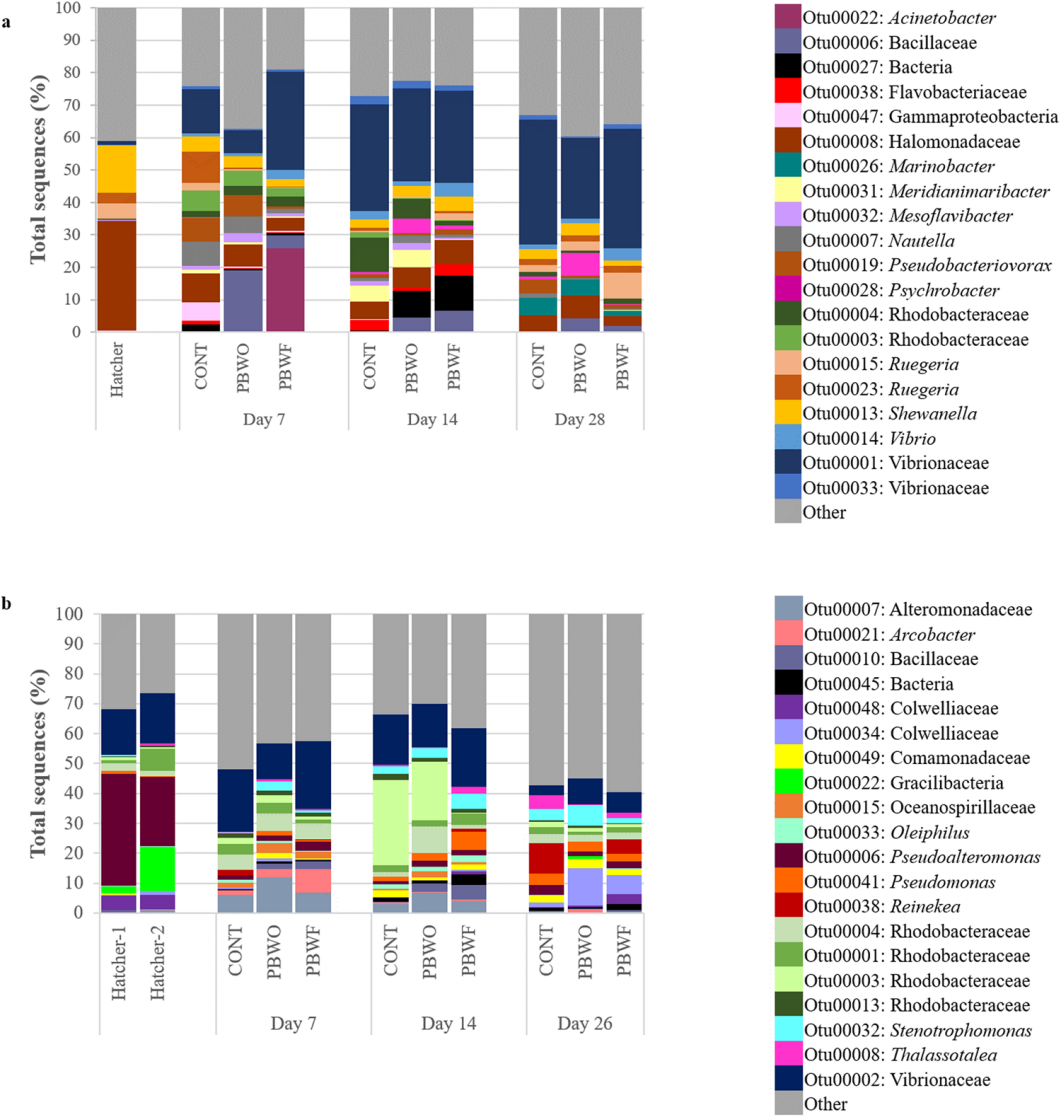
Relative abundances of the 20 most abundant OTUs identified in common snook larvae during probiotic trials. (**a**) Trial 1; (**b**) Trial 2. Hatcher, larvae sampled from the egg hatcher; CONT, control; PBWO, probiotics in water only; PBWF, probiotics in water and live feed.

The 20 most dominant OTUs of the fish microbiota are shown in Fig. 5. Again, Bacillaceae was higher in probiotic-treated fish, with relative abundances decreasing over time. Larvae harbored higher abundances of Vibrios that generally increased with fish age. Rhodobacters were abundant in both trials, but were in higher relative abundance in Trial 2 at all sampling points. Bacterial OTUs classified within taxa that contain potential pathogens include the Vibrios, *Acinetobacter*, Flavobacteriaceae, Halomonadaceae, *Psychrobacter*, *Shewanella*, Alteromonadaceae, *Pseudoalteromonas*, and *Pseudomonas*.

#### Treatment-based differences in microbiota

PERMANOVA results indicated a significant interaction between sample type (water vs fish), sampling day, and treatment on microbiota structure in both trials (Supplementary Table S1); however, there was generally more similarity between communities within Trial 2. For example, within water samples and on all days (Days 0, 7, 14, 28), water microbiota was significantly different among all treatments in Trial 1 (Supplementary Table S2), but in Trial 2 CONT was only different from PBWO at Day 7. Probiotic-treated fish had indiscriminant microbiota (PBWO vs PBWF) at 14 dph of Trial 1, while at 28 dph, the microbiota of PBWF fish was not different from that of CONT. The only significant difference between larval microbiota in Trial 2 was between CONT and PBWF at 7 dph.

Differences among specific OTUs were determined by LEfSe. In an attempt to elucidate the mechanism behind the probiotic benefits, only taxa that were discriminatory of a treatment in both trials are discussed. OTUs within 12 taxa were identified as consistently discriminatory between treatments in larvae (Supplementary Table S3). Bacillaceae, likely representing our probiotic strains, were increased in treatments as compared to CONT at 7 and 14 dph, but not at 28/26 dph. *Tenacibaculum* was higher in PBWO at 7 dph, whereas *Erythrobacter* was higher in PBWF on the same day. OTUs identified within the Rhodobacteraceae were often decreased in treatment larvae. More OTUs were differential between treatments from the water microbiota, falling into 40 taxa (Supplementary Table S4). Within these groups, it was not uncommon for some OTUs to be increased in CONT, and separate OTUs within the same taxon to be increased in treatments. Bacillaceae were enriched in treatment systems at all days except Day 14. *Idiomarina*, *Coxiella*, and *Alcanivorax* were higher in CONT at Day 0, whereas *Kangiella* was higher in treatments at Day 0. *Arcobacter* was higher in both treatments at Day 28/26 as compared to CONT.

#### Predicted microbiota function

NSTI scores averaged 0.096 ± 0.035 (mean ± SD) in Trial 1 and 0.086 ± 0.005 in Trial 2, indicating accurate predicted microbial community function (an NSTI score of 0.17 was found to provide accurate metagenome predictions in soil samples^23^). Pathways within xenobiotics biodegradation and metabolism were higher in probiotic-treated larvae than CONT at 7 dph, including dioxin, xylene degradation, ethylbenzene, and styrene degradation (Supplementary Table S5). In addition, sporulation was higher in PBWF than CONT at 14 dph. Greater similarities were seen between predicted microbiota function in water communities. A majority of altered functions were seen at Days 0 and 7, with most enriched pathways found within metabolism, and a majority of these were enriched in probiotic-treated larvae. One pathway associated with xenobiotics biodegradation was higher in treated water. Pathways associated with the sulfur cycle were enriched in CONT systems.

## Discussion

This study demonstrated the benefits of probiotic supplementation to larval rearing in common snook, with significantly higher survival and resistance to transport stress in probiotic-treated larvae as compared to controls. These benefits could not be explained by faster larval growth. In fact, CONT larvae were significantly longer than probiotic-treated larvae, likely due to decreased competition for food in CONT tanks which exhibited significantly lower survival. The other differing morphometric in this study was oil globule volume which was lowest in CONT larvae, suggesting that CONT larvae were consuming their endogenous reserves more quickly than probiotic-treated larvae. Retention of oil globules allows for a longer transition time to exogenous feeding, and studies indicate larvae that retain their endogenous reserves longer demonstrate increased survival^25,26^. The probiotic may alter development of the digestive tract and thus the start of exogenous feeding, as has been demonstrated in previous studies involving *Bacillus* probiotics and common snook^27^. Histological studies should be done in future experiments to explore this possibility.

The increased survival of probiotic-treated larvae following transport is notable, as the probiotic continued to exert its effects after supplementation ended. The mechanism behind this protection could not be identified by data collected in these studies. There were no significant differences in measured innate immune enzyme activities (LYS, SOD, ALP) in either Trial, but it is possible that the probiotic was positively influencing the fish immune system in parameters not measured here. Studies using *Bacillus* spp. as probiotics have demonstrated improvements in immune function and disease resistance in various fish species^12,28–31^. These responses should be further explored using in-depth immune assays, as well as transcriptomics.

Administration of the probiotic may have increased disease resistance in snook, either through alterations in the immune system as discussed above, or through direct antagonism with pathogens present in the rearing systems. Indeed, *Bacillus* probiotics are reported to suppress fish pathogens including Vibrios^12,32^. Vibrios were consistently present in relatively high abundances within our systems, and these bacteria are commonly reported as a dominant component of the fish larval microbiota after the start of exogenous feeding^14,33,34^; thus a majority of the organisms within this taxon are likely commensal or perhaps mutualistic. Unfortunately, 16S rRNA sequencing is not sufficient to classify some members of the Vibrionaceae to the species or strain level^35^ and future studies investigating the influence of Vibrios on larval survival should use techniques capable of distinguishing pathogenic species. Although the relative abundance of Vibrios was not significantly different between treatments, it is possible the probiotic inhibits colonization of pathogenic Vibrios in these systems.

Overall, survival during Trial 2 was higher than that of Trial 1, including that within CONT systems. A number of mechanisms for this decreased mortality are possible. The feeding protocols were altered between trials due to results from other experiments in the facility, and these protocols may have been better suited for larval rearing of common snook. Additionally, the bacterial community structure was more stable across treatments from the start (Day 0) in Trial 2 as compared to Trial 1. This is likely due to the systems circulating for five weeks prior to addition of larvae, allowing the microbial assemblages time to stabilize in an oligotrophic environment. This strategy favors proliferation of K-strategists which often contain commensal or mutualistic bacterial species and prevents dominance of r-strategists which often include pathogens and opportunists^36^. We hypothesize that the stabilized community was better able to prevent proliferation of opportunists once the newly hatched larvae were stocked into the tanks, allowing for a relatively stable microbiota over time and protecting larvae from detrimental host-microbe interactions. It was also noted that larvae from Trial 2 had decreased SOD activity coupled with increased LYS activity in all treatments, and this may indicate a reduction in oxidative stress and increased innate immune capacity in these larvae. The microbiota trains the immune system^37^, and exposure to a more diverse microbial community in Trial 2 (the top 20 OTUs make up less than 50% of the total sequences in Trial 2 versus 60–70% in Trial 1) may have improved the development of the larval immune system. Although an altered feeding regime was likely partially responsible for this overall improvement, we suggest that earlier stabilization of the microbial communities is also vital to larval survival in rearing systems.

Few OTUs were listed as differential consistently between Trials 1 and 2 within a treatment. Within the larval microbiota, only Bacillaceae was consistently enriched in probiotic-treated larvae in both trials. Due to the lack of this OTU in the CONT systems, we hypothesize this OTU is associated with our probiotic strains, and we are in the process of developing quantitative PCR to detect and quantify the probiotic within our systems. Differential taxa within the water microbiota included three genera enriched in CONT systems: *Idiomarina*, *Coxiella*, and *Alcanivorax*. All three of these groups are associated with water in aquaculture^38,39^, particularly the biofilters in recirculating systems^40,41^. *Idiomarina* has been reported in the microbiota of other fishes^42–45^ and may act as a normal inhabitant within culture systems. *Coxiella* is typically described as an intracellular pathogen capable of infecting a wide range of vertebrates and invertebrates^46,47^ and is rarely reported in fishes^48^, so its role in our systems is largely undetermined. The genus *Alcanivorax* degrades pristane, a terpenoid alkane, naturally produced by some zooplankton^49^. Reports in the literature suggest some strains of *B. licheniformis* and *B. amyloliquefaciens* are also capable of degrading this compound^50,51^, and the probiotic strains may be assuming this role in treated systems. Degradation of naturally-occurring hydrocarbons is associated with biosurfactant production, compounds that can interfere with bacterial communication (quorum sensing), leading to a reduction in virulence gene expression in fish pathogens^52^. Biosurfactants produced by *Bacillus* strains^53^ may harbor similar benefits for interfering with pathogenesis in probiotic-treated systems.

OTUs consistently enriched in probiotic-treated systems included *Kangiella* and *Arcobacter*. Both of these genera have been previously reported as dominant members of the rotifer microbiota^14,54,55^, and this genus is likely transferred to the water through addition of live feed. Species within *Kangiella* and *Arcobacter* both metabolize nitrogen compounds^56–58^ and may play a role in nitrogen cycling in aquaculture. An increase in these genera in probiotic-treated systems may indicate more efficient removal of nitrogenous fish waste from the system.

The similarities in performance between the trials, despite few similarities in microbiota taxonomical structure, suggest a level of functional redundancy within these systems. Within the larval microbiota, sporulation was enriched in PBWF as compared to CONT in both trials, a function likely associated with the endospores of the probiotic. However, it is not clear why this pathway was not enriched in PBWO. Within the xenobiotics biodegradation and metabolism pathway, probiotic-treated larvae harbored greater ability to degrade xenobiotics, although the specific xenobiotics differed between trials. The greater xenobiotic degradation capability of microbiota associated with probiotic-treated larvae may contribute to greater larval survival.

The majority of KEGG pathways enriched in the water were found early in the trials (Days 0 and 7), suggesting the water microbiota at initial larval stocking may be essential to the success demonstrated with probiotic supplementation. In fact, pathways increased in CONT water are typically associated with pathogenesis (bacterial chemotaxis^59,60^) and poor water quality (sulfur relay system, sulfur metabolism, porphyrin and chlorophyll metabolism), whereas those higher in treated water are associated with energy (production of short chain fatty acids, SCFA) and increased immunity. Bacterial chemotaxis may be involved in virulence for opportunists in the system or perhaps in biofilm establishment, providing a reservoir for harmful microbes^61^. The increase in sulfur-related pathways in CONT systems may indicate a higher abundance of available sulfur in the water which is potentially problematic as various forms of sulfur can be toxic to fish^62–65^. Future studies should quantify sulfur during daily water quality measurements. Porphyrins are often used as indicators of environmental pollution^66,67^; thus increase in their metabolism in CONT tanks may be indicative of water quality differences between treatments.

Pathways elevated in probiotic-treated water included thirteen pathways within metabolism. The microbiota of treated water had greater ability to degrade lysine, an amino acid precursor to beneficial compounds such as carnitine and glutamate/glutamine. These products aid in growth, protect against ammonia and xenobiotics, enhance the stress response, and act as energy sources for immune and other cells in fishes^68^. Other bacterial functions that suggest an increase in energy sources for fish cells include butanoate, proponoate, and pyruvate metabolism, synthesis and degradation of ketone bodies, and pantothenate and CoA biosynthesis. The increase in these pathways suggests the water microbiota may be providing energy benefits to the fish larvae upon stocking of the systems. Probiotic-treated water also included numerous upregulated pathways associated with immune health, including peroxisome which protects cells against oxidative stress^69^, and terpenoid backbone synthesis and geraniol degradation whose benefits include antioxidant, anti-inflammatory, and wound healing capabilities^70,71^. Additionally, phosphonate and phosphinate metabolism was increased. These antimicrobial compounds are produced by some members of *Bacillus*^72^. These differences in early water communities indicate that addition of probiotic alters bacterial community structure to one more suited for larval success, supporting the hypothesis that bacterial management is vital to larval production; however, it may be more important to focus on the role of these microbes in the aquaculture system than on the specific taxa present.

In conclusion, use of a mixed *Bacillus* species probiotic improves survival and transport stress resistance in common snook. Data from two trials suggest that potential mechanisms for larval rearing improvement include enhanced development of the gastrointestinal tract, boosted immunity, inhibition of pathogens and opportunists, and improved water quality. Early stabilization of microbiota within RAS improved overall success in fish production, and also improved performance of the probiotic. The range of benefits provided by these *Bacillus* strains suggests the potential for this probiotic to be valuable in other fish species, improving the sustainability of recirculating aquaculture and reducing the larval rearing bottleneck to enhance the overall efficiency of fish production systems.

## Supplementary information

Supplementary Tables

## Data Availability

All sequencing data has been submitted to the Sequence Read Archive (https://www.ncbi.nlm.nih.gov/sra) under the following SRA identifiers: Trial 1 larvae-associated sequences, SRP148994; Trial 1 water-associated sequences, SRP149333; Trial 2 larvae-associated sequences, SRP149334; Trial 2 water-associated sequences, SRP149332.
