# Supplementary material for: Benefits of a Bacillus probiotic to larval fish survival and transport stress resistance
Source: Sci Rep. 2019 Mar 20;9:4892. doi: 10.1038/s41598-019-39316-w (PMC6426941; doi:10.1038/s41598-019-39316-w)
Supplement: Supplementary file 1 — Supplementary Tables [file 41598_2019_39316_MOESM1_ESM.pdf]

Manuscript Title: Benefits of a *Bacillus* probiotic to larval fish survival and transport stress resistance

Authors: Andrea M. Tarnecki, Marzie Wafapoor, Remy N. Phillips, Nicole R. Rhody

Supplemental Information

Supplementary Table S1. Permutational analysis of variance (PERMANOVA) results for microbiota structure between sample types (water versus larvae), sampling day, and treatment.

	Trial 1						Trial 2					
**Source**	**df**	**SS**	**MS**	**Pseudo-F**	**P(perm)**	**Unique permutations**	**df**	**SS**	**MS**	**Pseudo-F**	**P(perm)**	**Unique permutations**
Sample type	1	43,427.0	43,427.0	41.644	0.001	998	1	38,419.0	38,419.0	32.655	0.001	997
Day	2	43,692.0	21,846.0	20.949	0.001	998	2	32,349.0	16,174.0	13.748	0.001	996
Treatment	2	20,659.0	10,330.0	9.9053	0.001	998	3	9,984.1	3,328.0	2.8287	0.001	996
Sample type x Day	2	41,960.0	20,980.0	20.118	0.001	994	2	23,703.0	11,851.0	10.073	0.001	998
Sample type x Treatment	2	8,619.4	4,309.7	4.1327	0.001	999	2	3,730.9	1,865.5	1.5856	0.006	996
Day x Treatment	6	30,785.0	5,130.9	4.9201	0.001	998	6	18,967.0	3,161.2	2.6869	0.001	998
Sample type x Day x Treatment	4	15,674.0	3,918.5	3.7575	0.001	997	4	10,867.0	2,716.7	2.3091	0.001	996
Residuals	103	1.07E+05	1,042.8				109	1.28E+05	1,176.5			
Total	125	4.17E+05					133	3.99E+05				

Supplementary Table S2. Permutational analysis of variance (PERMANOVA) pair-wise comparison results for microbiota structure between treatments within each sampling type (water versus larvae) and sampling day. dph, days post hatch. Significant ($P < 0.05$) comparisons are shown in bold type.

		Trial 1			Trial 2		
**Sample Type**	**Day**	**CONT vs PBWO**	**CONT vs PBWF**	**PBWO vs PBWF**	**CONT vs PBWO**	**CONT vs PBWF**	**PBWO vs PBWF**
Fish	7 dph	**0.007**	**0.028**	**0.003**	0.200	**0.031**	0.474
	14 dph	**0.008**	**0.003**	0.119	0.157	0.339	0.053
	28/26 dph	**0.028**	0.145	**0.017**	0.181	0.615	0.078
Water	Day 0	**0.005**	**0.003**	**0.006**	0.080	**0.004**	**0.003**
	Day 7	**0.002**	**0.002**	**0.003**	**0.007**	**0.016**	**0.004**
	Day 14	**0.036**	**0.002**	**0.005**	0.774	**0.003**	**0.010**
	Day 28/26	**0.004**	**0.006**	**0.009**	0.072	**0.009**	**0.011**

Supplementary Table S3. Number of consistently discriminative OTUs (as determined by linear discriminant analysis effect size, LEfSe[24]) among treatments within the fish microbiota during two probiotic trials. Red indicates higher abundances in control larvae; green indicates higher abundances in probiotic-treated larvae.

	Day 7				Day 14				Day 28/26			
	PBWO		PBWF		PBWO		PBWF		PBWO		PBWF	
**Taxon**	**Trial 1**	**Trial 2**	**Trial 1**	**Trial 2**	**Trial 1**	**Trial 2**	**Trial 1**	**Trial 2**	**Trial 1**	**Trial 2**	**Trial 1**	**Trial 2**
Domain Bacteria	1	3	0	0	0	0	0	0	0	0	0	0
Genus *Tenacibaculum*	1	1	0	0	0	0	0	0	0	0	0	0
Family Bacillaceae	1	1	1	1	1	2	1	2	0	0	0	0
Phylum Proteobacteria	0	0	0	0	0	0	0	0	0	0	1	1
Order Alphaproteobacteria Incertae Sedis	1	5	1	7	0	0	0	0	0	0	0	0
Family Rhodobacteraceae	0	0	1	8	0	0	3	1	4	1	0	0
Genus *Erythrobacter*	0	0	1	2	0	0	0	0	0	0	0	0
Class Deltaproteobacteria	0	0	0	0	0	0	0	0	0	0	1	1
Class Gammaproteobacteria	2	8	3	8	0	0	0	0	1	2	2	4
Family Colwelliaceae	0	0	0	0	0	0	0	0	0	0	1	1
Family Halieaceae	0	0	0	0	0	0	0	0	0	0	1	1
Phylum TM6 (Dependentiae)	0	0	1	1	0	0	0	0	0	0	0	0

Supplementary Table S4. Number of consistently discriminative OTUs (as determined by linear discriminant analysis effect size, LEfSe[24]) among treatments within the water microbiota during two probiotic trials. Red indicates higher abundances in control larvae; green indicates higher abundances in probiotic-treated larvae. Where some OTUs were higher in control larvae and other OTUs within the same taxon were higher in treatment larvae, the cell is split appropriately.

	Day 0				Day 7				Day 14				Day 28/26			
	PBWO		PBWF		PBWO		PBWF		PBWO		PBWF		PBWO		PBWF	
Taxon	Trial 1	Trial 2	Trial 1	Trial 2	Trial 1	Trial 2	Trial 1	Trial 2	Trial 1	Trial 2	Trial 1	Trial 2	Trial 1	Trial 2	Trial 1	Trial 2
Domain Bacteria	1 / 1	1 / 6	1 / 1	13 / 7	0	0	1	2	3	5	2	2	1 / 7	3 / 3	1 / 8	5 / 4
Order Flavobacteriales	1	1	0	0	0	0	0	0	0	0	0	0	0	0	0	0
Family Flavobacteriaceae	2	1	2	1	0	0	0	0	0	0	1	1	0	0	0	0
Genus *Tenacibaculum*	0	0	0	0	2	1	0	0	0	0	0	0	0	0	0	0
Family NS11-12 marine group	3	1	1	1	0	0	0	0	0	0	0	0	0	0	0	0
Order Chlamydiales	1 / 1	7 / 4	1	10	0	0	0	0	0	0	0	0	0	0	0	0
Family Bacillaceae	1	1	1	1	1	1	1	1	0	0	0	0	1	1	1	1
Genus *Fusibacter*	0	0	0	0	0	0	0	0	0	0	0	0	0	0	1	1
Phylum Gracilibacteria	1	1	1 / 1	1 / 2	0	0	0	0	1	1	1	2	4	2	7	2
Class Pacebacteria	0	0	0	0	0	0	0	0	0	0	2	1	0	0	1	1
Phylum Parcubacteria	0	0	0	0	0	0	0	0	0	0	0	0	0	0	1	5
Phylum Peregrinibacteria	0	0	0	0	0	0	4	3	0	0	0	0	5	2	2	2
Genus *Planctomyces*	1	1	0	0	0	0	0	0	0	0	0	0	0	0	0	0
Phylum Proteobacteria	1 / 1	3 / 3	0	0	1	3	1 / 1	2 / 3	0	0	4	2	1 / 8	2 / 1	1 / 2	1 / 2
Class Alphaproteobacteria	4	5	2 / 3	4 / 4	0	0	1	2	1	1	5	2	0	0	4 / 1	1 / 1
Order 4-Org1-14	1	3	0	0	0	0	1	1	0	0	0	0	0	0	0	0
Order Alphaproteobacteria Incertae Sedis	3 / 1	3 / 4	1	2	1	3	2	5	3	1	18	4	15	1	1 / 15	4
Family Erythrobacteraceae	1	1	0	0	0	0	0	0	0	0	0	0	0	0	0	0
Order Rhizobiales	0	0	1 / 2	1 / 1	0	0	0	0	0	0	0	0	0	0	0	0
Genus *Hyphomicrobium*	0	0	1	1	0	0	0	0	0	0	0	0	0	0	0	0
Genus *Maritalea*	0	0	0	0	0	0	0	0	0	0	0	0	1	1	0	0
Family OCS116 clade	0	0	1	1	0	0	0	0	0	0	0	0	0	0	0	0
Family Rhodobacteraceae	5	4 / 7	7 / 4	7 / 5	5	5	1 / 5	2 / 1	0	0	2 / 2	2 / 3	2 / 3	4 / 2	3 / 3	3 / 1
Genus *Epibacterium*	0	0	0	0	0	0	0	0	0	0	0	0	0	0	1	1
Family Rhodospirillaceae	2	1	1	3	0	0	0	0	0	0	0	0	0	0	0	0
Order Rickettsiales	0	0	1	2	0	0	0	0	0	0	0	0	0	0	2	1
Class Deltaproteobacteria	0	0	0	0	0	0	0	0	0	0	0	0	2	1	0	0
Genus *Arcobacter*	0	0	0	0	0	0	0	0	0	0	0	0	1	1	2	1
Class Gammaproteobacteria	11 / 5	13 / 6	6 / 7	15 / 9	3	2	3 / 5	1 / 5	9	4	11	3	4 / 1	7 / 3	13 / 14	4 / 3
Genus *Marinobacter*	0	0	0	0	0	0	0	0	1	1	0	0	0	0	0	0
Genus *Idiomarina*	2	2	3	2	0	0	0	0	0	0	0	0	0	0	0	0
Genus *Coxiella*	2	1	1	1	0	0	0	0	0	0	0	0	0	0	0	0
Family Legionellaceae	1	1	0	0	0	0	0	0	0	0	0	0	0	0	0	0
Genus *Legionella*	0	0	0	0	0	0	0	0	0	0	1	1	0	0	0	0
Genus *Alcanivorax*	1	1	1	1	0	0	0	0	0	0	0	0	0	0	0	0
Genus *Kangiella*	1	1	1	1	0	0	0	0	0	0	0	0	0	0	0	0
Genus *Halomonas*	1	1	0	0	0	0	0	0	0	0	1	1	0	0	0	0
Family Vibrionaceae	0	0	0	0	0	0	0	0	0	0	1	3	0	0	0	0
Phylum SBR1093	0	0	2	1	0	0	0	0	0	0	0	0	0	0	0	0
Phylum TM6 (Dependentiae)	3 / 1	4 / 3	1 / 4	4 / 3	0	0	0	0	0	0	5 / 4	1 / 1	0	0	0	0

Supplementary Table S5. Consistent differences in predicted metagenome function (as determined by phylogenetic investigation of communities by reconstruction of unobserved states, PICRUSt[23]) among treatments within the fish and water microbiota during two probiotic trials. The treatment with the highest level of function is listed for each Trial. Functions increased in control are highlighted in gray. Functions increased in probiotic-treatments are highlighted in green. N/A indicates no significant difference within a KEGG pathway in a Trial.

Sample Type	Level 1	Level 2	Level 3	Day	Trial 1	Trial 2
Fish	Cellular Processes	Sporulation	unclassified	14	PBWF	PBWF
	Metabolism	Xenobiotics biodegradation and metabolism	Dioxin degradation	7	PBWO/PBWF	N/A
	Metabolism	Xenobiotics biodegradation and metabolism	Ethylbenzene degradation	7	N/A	PBWO
	Metabolism	Xenobiotics biodegradation and metabolism	Styrene degradation	7	N/A	PBWO
	Metabolism	Xenobiotics biodegradation and metabolism	Xylene degradation	7	PBWF	N/A
Water	Cellular Processes	Cell motility	Bacterial chemotaxis	0	CONT	N/A
	Cellular Processes	Transport and catabolism	Peroxisome	7	PBWO/PBWF	PBWF
	Genetic Information Processing	Folding, sorting and degradation	Sulfur relay system	0	CONT	CONT
	Genetic Information Processing	Translation	Aminoacyl-tRNA biosynthesis	7	PBWO/PBWF	PBWO > CONT
	Metabolism	Amino acid metabolism	Lysine degradation	0	PBWO/PBWF	PBWO/PBWF
	Metabolism	Biosynthesis of other secondary metabolites	Isoflavonoid biosynthesis	0	PBWO/PBWF	PBWO/PBWF
	Metabolism	Biosynthesis of other secondary metabolites	Isoflavonoid biosynthesis	28/26	PBWO/PBWF	PBWO/PBWF
	Metabolism	Carbohydrate Metabolism	Butanoate metabolism	0	PBWO/PBWF	PBWO/PBWF
	Metabolism	Carbohydrate Metabolism	Propanoate metabolism	0	PBWO/PBWF	PBWO/PBWF
	Metabolism	Carbohydrate Metabolism	Pyruvate metabolism	7	PBWF	PBWF
	Metabolism	Energy metabolism	Sulfur metabolism	0	CONT	CONT
	Metabolism	Lipid metabolism	Synthesis and degradation of ketone bodies	0	PBWO/PBWF	PBWO/PBWF
	Metabolism	Metabolism of cofactors and vitamins	Pathothenate and CoA biosynthesis	7	PBWO/PBWF	PBWF
	Metabolism	Metabolism of cofactors and vitamins	Porphyrin and chlorophyll metabolism	0	CONT	N/A
	Metabolism	Metabolism of cofactors and vitamins	Vitamin B6 metabolism	0	CONT	CONT
	Metabolism	Metabolism of other amino acids	Phosphonate and phosphinate metabolism	28/26	PBWF	PBWF
	Metabolism	Metabolism of terpenoids and polyketides	Geraniol degradation	0	PBWO/PBWF	PBWO/PBWF
	Metabolism	Metabolism of terpenoids and polyketides	Geraniol degradation	7	PBWF	PBWF
	Metabolism	Metabolism of terpenoids and polyketides	Terpenoid backbone biosynthesis	7	PBWF	PBWO/PBWF
	Metabolism	Xenobiotics biodegradation and metabolism	Naphthalene degradation	0	PBWO/PBWF	PBWO/PBWF
